# Shexiang Huayu Xingnao Granule Alleviates Intracerebral Hemorrhage via PI3K/AKT‐Mediated Neuroinflammation Regulation: Integrated Network Pharmacology and Experimental Validation

**DOI:** 10.1002/brb3.71853

**Published:** 2026-10-30

**Authors:** Yuan RuKui, Wang MingLi, Li Chun, Chen MeiJuan, Yuan Yuan

**Affiliations:** ^1^ The Affiliated Traditional Chinese Medicine Hospital of Southwest Medical University Luzhou China; ^2^ Compounded Infusion Quality Testing Analysis and Precision Medication Technological Innovation Key Laboratory of Luzhou Luzhou China; ^3^ School of Pharmacy Southwest Medical University Luzhou China

**Keywords:** intracerebral hemorrhage, network pharmacology, neuroinflammation, PI3K/AKT pathway, Shexiang Huayu Xingnao granules, traditional Chinese medicine

## Abstract

**Objective:**

Employing network pharmacology prediction combined with experimental validation, this research elucidated the underlying mechanisms of Shexiang Huayu Xingnao granules (SXHY) against intracerebral hemorrhage (ICH).

**Methods:**

Potential targets and related signaling pathways of SXHY acting on ICH were predicted through network pharmacology. An autologous blood injection‐induced rat ICH model was established, with simvastatin as the positive control drug. The therapeutic effects of SXHY were evaluated using neurological function scores, brain water content, superoxide dismutase (SOD), catalase, malondialdehyde activity, and brain tissue hemorrhage area. Mechanism studies used a lipopolysaccharide‐stimulated BV‐2 microglial cell model, assessing inflammatory mediator expression profiles and PI3K/AKT pathway modulation through RT‐qPCR and Western blot analysis.

**Results:**

Network pharmacology analysis revealed 360 common SXHY‐ICH targets, with 10 core targets (including TNF, AKT1, IL‐6, and IL‐1β) significantly enriched in the PI3K/AKT, AGE‐RAGE, and MAPK pathways; molecular docking further demonstrated potent binding of the key component stigmasterol to TNF‐α (binding energy: −9.125 kcal/mol) and IL‐6 (binding energy: −8.57 kcal/mol). Animal experiments showed within‐group improvements in neurological function from day 2 to day 3 post‐ICH in SXHY groups. The observed proportions of animals with improved neurological scores were 88.9% (low‐dose), 83.3% (high‐dose), 70.0% (model), and 62.5% (simvastatin). However, no statistically significant between‐group differences were observed at day 3 (*p* > 0.05). It also reduced brain edema (high dose: 78.0 ± 0.7% vs. model: 80.5 ± 1.0%, *p <* 0.01) and significantly decreased hematoma area (high dose: 0.35 ± 0.50 mm^2^ vs. model: 1.02 ± 0.93 mm^2^, *p <* 0.05); meanwhile, it significantly increased SOD activity (high dose: 68.7 ± 11.3 U/mg protein vs. model: 37.3±14.1 U/mg protein, *p <* 0.01). Cell experiments further confirmed that SXHY mediates anti‐inflammatory activity through modulation of PI3K/AKT signaling, including reduced p‐PI3K/GAPDH (*p* < 0.01), downregulation of proinflammatory cytokines TNF‐α/IL‐6/IL‐1β mRNA (*p* < 0.01), and upregulation of anti‐inflammatory cytokine IL‐10 mRNA (*p* < 0.05).

**Conclusion:**

SXHY may alleviate post‐ICH brain injury by inhibiting neuroinflammation throu modulation of the PI3K/AKT pathway. The results provide new mechanistic evidence for traditional Chinese medicine interventions against ICH.

## Graphical abstract

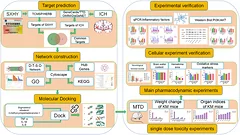

## 1 Introduction

Intracerebral hemorrhage (ICH), resulting from cerebrovascular rupture leading to blood extravasation into brain parenchyma, represents an acute brain injury characterized by sudden onset, rapid progression, and high mortality/disability rates (Greenberg et al. 2022; Tsao et al. 2022). Epidemiological evidence confirms that ICH contributes to approximately 28.8% of global stroke cases, exhibiting a disproportionately elevated burden characterized by increased incidence, mortality, and long‐term disability rates in low‐ and middle‐income countries relative to high‐income Western nations (Feigin et al. 2025; Wolsink et al. 2024). ICH not only poses severe threats to individual health but also imposes substantial economic and caregiving burdens. Current clinical management primarily relies on Western medical approaches, including antihypertensive therapy, intracranial pressure control, surgical hematoma evacuation, and supportive care. However, the lack of definitive targeted interventions results in modest prognostic improvement.

Traditional Chinese medicine (TCM) constitutes an indispensable element of China's healthcare framework, with evidenced unique advantages in preventing and treating neurological disorders, particularly through the provision of innovative therapeutic strategies against ICH via multi‐target mechanisms and holistic regulation (Yin et al. 2025; Chen and Jin 2023).

Shexiang Huayu Xingnao granules (SXHY) were developed based on TCM theories of “the brain housing the primordial spirit” and “wind pathogen‐induced pathogenesis,” following the principle of “resolving stasis to unblock collaterals and dispelling wind to restore consciousness.” Composed of six ingredients, including *Moschus*, *Astragali* Radix, and *Panax Notoginseng*, SXHY exerts multi‐target effects: *Moschus* (primary component) penetrates the blood–brain barrier to inhibit the TNF‐α/RIPK1 necroptotic pathway, downregulating AQP4 and HIF‐1α expression, thereby alleviating neuroinflammation and brain edema (Han et al. 2025); Astragaloside IV, a key bioactive compound in *Astragali Radix*, upregulates neurotrophic factors to promote neurorepair and suppresses P‐Src/P‐GRK2 phosphorylation, protecting brain cells from oxidative damage post‐ICH (Chen et al. 2024; Yao et al. 2023); *Notoginseng* saponins enhance meningeal lymphatic clearance of inflammatory mediators, accelerating hematoma resolution (Yu et al. 2024). Clinical evidence confirms that herbal formulas containing *Moschus* and *Panax Notoginseng* improve neurological recovery and hematoma absorption in ICH patients (Song et al. 2024; Guo et al. 2024), supporting SXHY's efficacy as an adjuvant therapy.

This study integrated network pharmacology prediction, animal model validation, and molecular mechanistic exploration to evaluate the safety, efficacy, and mechanisms of SXHY against ICH, providing experimental evidence for TCM‐based multi‐target regulation of ICH. Figure 1 displays the study’s flowchart.

## 2 Materials and Methods

### 2.1 Prediction of Active Components and Targets in SXHY

The chemical constituents of six herbal components in SXHY (*Moschus*, *Astragali* Radix, *Cinnamomi Ramulus*, *Alismatis Rhizoma*, *Notoginseng Radix*, and *Rhei Radix et Rhizoma*) were retrieved from the TCM Systems Pharmacology Database (TCMSP, v2.3, https://old.tcmsp‐e.com/) under ADME criteria: oral bioavailability ≥30% and drug‐likeness ≥0.18 (Jia et al. 2020). Components not in TCMSP were supplemented via HERB (http://herb.ac.cn/). Canonical SMILES were imported into Swiss Target Prediction (http://www.swisstargetprediction.ch/; probability ≥0.1; *Homo sapiens*). Merged data were deduplicated to construct the component‐target dataset.

### 2.2 Acquisition of ICH‐Related Genes

To analyze intersections between herbal component targets and disease targets, ICH‐associated human genes were systematically retrieved from GeneCards (v5.24; https://www.genecards.org/), OMIM (https://omim.org/), TTD (http://db.idrblab.net/ttd/), and DisGeNET (v24.4; https://www.disgenet.org/) using the keywords “Intracerebral hemorrhage” AND “Hemorrhagic stroke.” The intersecting targets between SXHY components and ICH genes were identified via Venny 2.1 (https://bioinfogp.cnb.csic.es/tools/venny/) and visualized as a Venn diagram highlighting pharmacologically and pathologically significant shared targets.

### 2.3 Construction of PPI Network and Core Target Screening

To investigate protein interactions of core targets, shared target genes were imported into the STRING database (v11.5; https://string‐db.org/) under parameters: species “Homo sapiens,” minimum interaction confidence >0.9 (high confidence), and disconnected nodes hidden to construct the protein–protein interaction (PPI) network. The network was visualized using Cytoscape (v3.10.2). Core targets were screened through topological analysis with the CytoNCA plugin, evaluating both centrality metrics (degree [DC], betweenness [BC], closeness [CC]) and network robustness parameters (eigenvector [EC], local average connectivity [LAC], network centrality [NC]). The dual‐threshold screening criteria were DC ≥ median value and BC ≥ top 20%.

### 2.4 Gene Ontology and KEGG Pathway Enrichment Analysis

The functional annotation of shared targets was carried out using DAVID 6.8 (https://david.ncifcrf.gov/). In the Gene Ontology (GO) enrichment analysis, which includes biological process (BP), molecular function (MF), and cellular component (CC), a Benjamini–Hochberg adjusted *p* < 0.05 was set as the significance criterion. For the Kyoto Encyclopedia of Genes and Genomes (KEGG) pathway analysis, an additional criterion was applied: only pathways with a nominal *p* < 0.01 and containing more than 5 target genes were deemed significantly enriched. The top 30 significantly enriched BP and signaling pathways were visualized using bubble plots.

### 2.5 Multidimensional Network Construction

To systematically elucidate the mechanisms of SXHY intervention in ICH, a multidimensional visualization network integrating four hierarchical layers was constructed: (1) Active compound layer: Bioactive components screened from TCMSP/HERB databases; (2) Target layer: Shared drug–disease targets identified via Venny; (3) Pathway layer: Top 30 ICH‐associated signaling pathways (KEGG enrichment); and (4) Disease layer: Core ICH phenotypes (cerebral edema, hematoma expansion). Layers were merged using the “Merge” function in Cytoscape (v3.10.2) and topologically optimized with an edge‐weighted force‐directed layout. Network topology properties (average degree value and clustering coefficient) were quantitatively analyzed to evaluate multi‐target synergistic effects.

### 2.6 Molecular Docking Validation

To investigate binding modes between key bioactive components (top‐4 DC‐ranked) and core targets, molecular docking was performed. Ligands were prepared by converting 3D structures (PubChem) to PDBQT format (OpenBabel) with Gasteiger charge assignment; receptors were obtained from PDB (TNF‐α: 6NCF at 2.5 Å; IL‐6: 1ALU at 2.1 Å; IL‐1β: 4GNP at 2.0 Å) and prepared by water removal and polar hydrogen addition; molecular docking simulations were executed using AutoDock Vina 1.2.0 (Dockeas platform) with a grid centered on active sites (30 × 30 × 30 Å^3^; 0.05 nm spacing); conformations with binding energy ≤ −5.0 kcal/mol and ≥2 hydrogen bonds were retained for validation, with key interactions visualized in PyMOL.

### 2.7 Reagents and Instruments

SXHY was provided by the Department of Pharmacy, Affiliated Traditional Chinese Medicine Hospital of Southwest Medical University (Batch: 20230510). Simvastatin tablets were supplied by Hangzhou MSD Pharmaceutical Co., Ltd. (Batch: X025657). Commercial assay kits including catalase (CAT), malondialdehyde (MDA), and superoxide dismutase (SOD) activity detection kits, along with total protein quantification kits, were purchased from Nanjing Jiancheng Bioengineering Institute (Nanjing, China). Additional reagents include lipopolysaccharide (LPS) (Solarbio, Beijing), TRNzol Universal RNA reagent (TIANGEN, Beijing), RT‐PCR kit (Vazyme, Shanghai), PI‐103 (J&K Scientific, Beijing), YS‐49 (Aladdin, Shanghai), antibodies against total AKT1/2/3 (Affinity Biosciences, Jiangsu, AF6261), phospho‐AKT1/2/3 (Ser473) (Affinity Biosciences, Jiangsu, AF0016), total PI3K (Affinity Biosciences, Jiangsu, AF6242), phospho‐PI3K (Affinity Biosciences, Jiangsu, AF3242), GAPDH (Proteintech, Wuhan,10494‐1‐AP), goat anti‐rabbit IgG‐HRP (Proteintech, Wuhan, SA00001‐2), chloral hydrate (Kelong Chemical, Chengdu), formaldehyde (Kelong Chemical), and bone wax (Johnson & Johnson, Shanghai). Instruments used were stereotaxic apparatus (STOELTING, Shanghai) and microsyringes (Anting, Shanghai) for animal experiments; Western blot system, multimode microplate reader (Molecular Devices, USA), and thermal cycler (Bio‐Rad, USA) for cellular experiments.

### 2.8 ICH Animal Model

All experimental procedures involving SPF‐grade Sprague–Dawley (SD) rats (obtained from the Experimental Animal Center of Sichuan Academy of Traditional Chinese Medicine) were approved by the Institutional Animal Ethics Committee of Sichuan Academy of Traditional Chinese Medicine (Approval No.: SYLL2021039; Date: October 2021). The ICH model was established using an autologous blood injection method adapted from Feng et al. (2023). Briefly, under chloral hydrate anesthesia (6% w/v, 5 mL/kg i.p.), rats were immobilized in a stereotactic frame. A burr hole was drilled at 0.5 ± 0.1 mm rostral to bregma and 3.0 ± 0.1 mm lateral to the midline sagittal suture. 50 µL of autologous blood was injected vertically (25 µL/min) into the caudate nucleus (6 mm depth) via a microsyringe, followed by a 10‐min needle retention period before suture. Sham‐operated controls underwent identical procedures, excluding blood injection. Neurological deficits were assessed 24 h post‐surgery using the Bederson scoring system (scores 1–3 indicated successful model establishment), with exclusion criteria set for animals showing extreme neurological impairment (score >3) or mortality.

### 2.9 Positive Control

Simvastatin was selected as the positive control due to its well‐documented neuroprotective effects in ICH animal models – statins (including simvastatin) mitigate secondary brain injury by reducing neuroinflammation, alleviating brain edema, and improving neurological function (Zi et al. 2021; Guo et al. 2025; Chen et al. 2020). Notably, statins modulate the PI3K/AKT pathway, which aligns with our study's focus on PI3K/AKT‐mediated inflammation regulation (Xue and Yong 2020).

### 2.10 Acute Toxicity Assessment

All animal experiments were performed with randomization and blinded outcome assessments. Following preliminary tests indicating an undetectable median lethal dose (LD_50_), an OECD Guideline 423‐compliant (OECD 2001) maximum tolerated dose (MTD) study was executed. Forty SPF‐grade KM mice (18–22 g, equal sex distribution) were allocated to two groups: the SXHY group (*n* = 20) administered a single oral gavage of SXHY solution (2.04 g crude drug/mL, 40 mL/kg, totaling 244.8 g crude drug/kg), and the control group (*n* = 20) administered purified water (40 mL/kg) via oral gavage. All mice underwent 12‐h fasting (water ad libitum) before dosing, received intensive monitoring for 4 h post‐dosing, and were subsequently observed daily for 14 days with body weights documented on days 1, 2, 3, 7, and 14.

### 2.11 Pharmacodynamic Evaluation

SXHY doses (2.45, 4.90, and 9.80 g crude drug/kg) were established as 3‐, 6‐, and 12‐fold the proposed clinical dose (0.816 g crude drug/kg) (Reagan‐Shaw et al. 2008). SD rats were stratified by body weight and randomly assigned to seven groups: normal control (untreated, *n* = 10), sham (needle insertion only, no blood injection, *n* = 10), model (vehicle‐treated, *n* = 10), SXHY low‐dose (*n* = 10), medium‐dose (*n* = 10), high‐dose (*n* = 10), and simvastatin (10 mg/kg, *n* = 10) groups. A parallel cohort (*n* = 6/group) was set for 3‐day dosing to assess brain water content and oxidative indices (SOD, MDA, CAT). All groups received oral gavage (10 mL/kg/day); the normal control, sham, and model groups were given an equal volume of pure water. The 7‐day cohort was used for neurological scoring and pathological examination.

### 2.12 Cell Culture

BV‐2 microglial cells (iCell Bioscience Inc., Shanghai, China) were maintained in DMEM/F12 medium at 37°C under 5% CO_2_.

### 2.13 Cell Grouping and Treatment

Six experimental cohorts were established: (1) Control (Normal culture medium); (2) LPS (2 µg/mL LPS); (3) SXHY‐L + LPS (Pretreated with 25 µg/mL SXHY for 2 h → 2 µg/mL LPS, 24 h); (4) SXHY‐M + LPS (Pretreated with 50 µg/mL SXHY for 2 h → 2 µg/mL LPS, 24 h); (5) SXHY‐H + LPS (Pretreated with 100 µg/mL SXHY for 2 h → 2 µg/mL LPS, 24 h); (6) Simvastatin + LPS (Pretreated with 10 µg/mL simvastatin for 2 h → 2 µg/mL LPS, 24 h). For PI3K/AKT pathway intervention, all treatment groups (excluding Control) were co‐administered either the PI3K/AKT inhibitor PI‐103 (10 µmol/L) or agonist YS‐49 (30 µmol/L).

### 2.14 RT‐qPCR Analysis

Total RNA was extracted using TRIzol reagent, reverse transcribed into cDNA, and amplified by qPCR with SYBR Green (primers in Table 1). Thermal cycling: 95°C for 10 min; 40 cycles of 95°C for 15 s, 60°C for 15 s, and 72°C for 60 s. Relative mRNA expression of TNF‐α, IL‐1β, IL‐6, and IL‐10 was calculated using the 2‐ΔΔCT method.

**TABLE 1:** RNA primer sequence.

Gene	Forward primer (5′ → 3′)	Reverse primer (5′ → 3′)
TNF‐α(*m*)	CCCTCACACTCAGATCATCTTCT	GCTACGACGTGGGCTACAG
Il‐6(*m*)	CCAAGAGGTGAGTGCTTCCC	CTGTTGTTCAGACTCTCTCCCT
Il‐1β(*m*)	GCAACTGTTCCTGAACTCAACT	ATCTTTTGGGGTCCGTCAACT
Il‐10(*m*)	GGTGAGAAGCTGAAGACCCTC	GCCTTGTAGACACCTTGGTCTT

### 2.15 Western Blot Analysis

Protein extraction was performed with RIPA lysis buffer, followed by quantification using the BCA method. Proteins (20 µg per lane) underwent SDS‐PAGE separation and electrophoretic transfer to nitrocellulose membranes. After 1‐h blocking (5% non‐fat milk, RT), the membranes were subjected to overnight incubation (4°C) with primary antibodies targeting PI3K (1:1000), *p*‐PI3K (1:1000), Akt (1:1000), *p*‐Akt (Ser473; 1:1000), and GAPDH (1:5000). Subsequent 1‐h incubation (RT) with HRP‐linked secondary antibodies (1:7500) preceded protein band detection via enhanced chemiluminescence (ECL).

### 2.16 Statistical Analysis

Data are expressed as mean ± SD. Normality was assessed using the Shapiro–Wilk test, and homogeneity of variances was confirmed by Levene's test. Data meeting both assumptions were analyzed by one‐way ANOVA with Tukey's post hoc test; non‐normally distributed or heteroscedastic data were analyzed using the Kruskal–Wallis test. Analyses were performed using GraphPad Prism 10.0 (GraphPad Software, USA). Significance was set at *p* < 0.05 for all tests.

**FIGURE 1:**
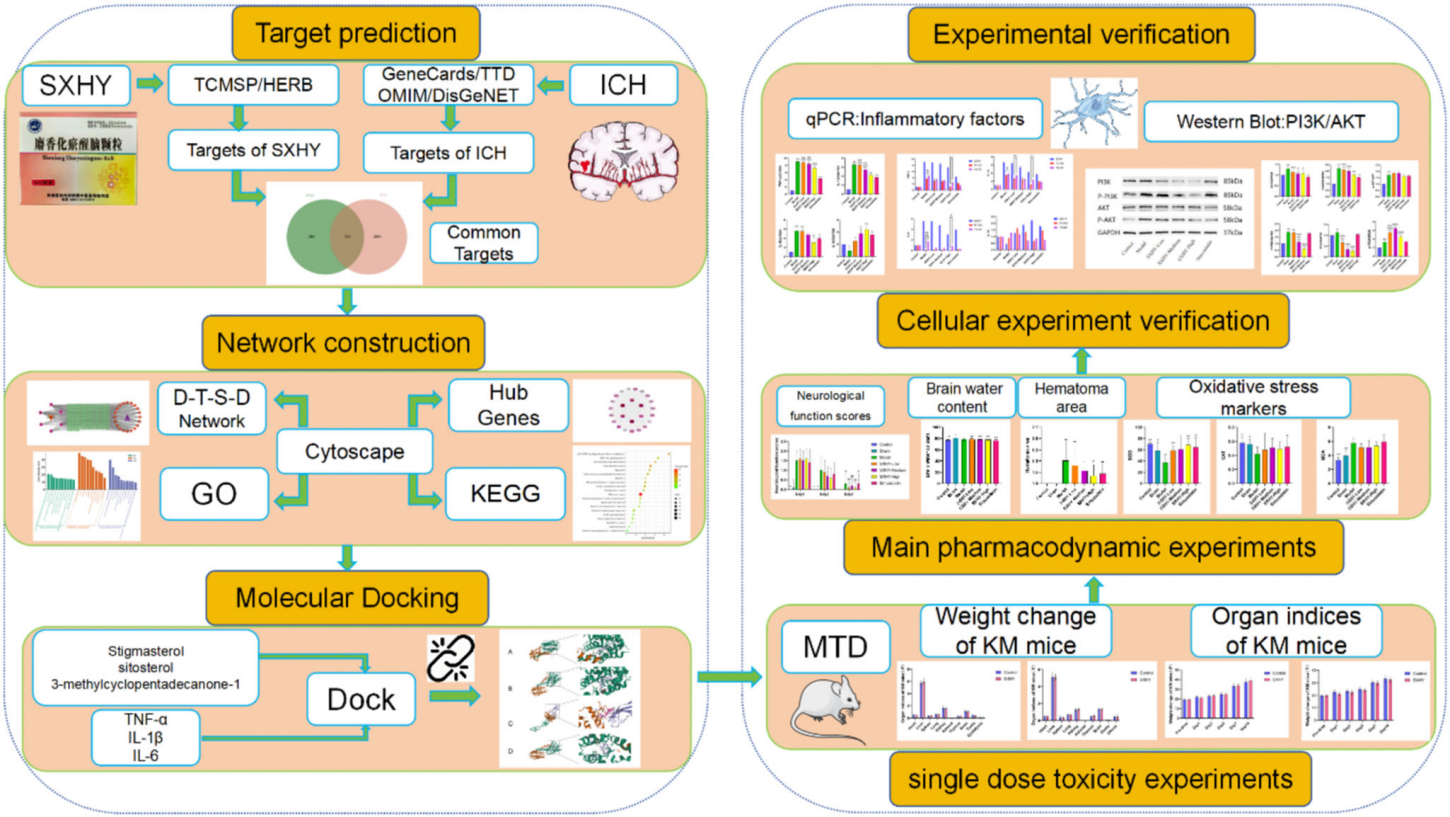
Graphical abstract.

## 3 Results

### 3.1 Integration of Network Pharmacology and Molecular Docking

Network pharmacology analysis identified 98 bioactive components in SXHY targeting 573 potential genes. Among 3274 ICH‐related targets, 360 overlapped with SXHY targets. PPI network analysis identified ten hub targets (TNF, AKT1, IL6, ALB, IL1B, TP53, SRC, CASP3, BCL2, STAT3), with TNF exhibiting the highest node degree. KEGG enrichment revealed SXHY's significant modulation of the PI3K/AKT pathway, AGE‐RAGE, and MAPK cascades. The 25 core hub genes interaction network and drug–target–signaling pathway–disease network suggest that SXHY might regulate ICH pathologies via the PI3K/AKT pathway (Figure 2). Molecular docking demonstrated strong binding between SXHY key components (e.g., stigmasterol) and core targets, particularly TNF‐α (binding energy = −10.18 kcal/mol) and IL‐6 (−8.66 kcal/mol) (Table 2 and Figure 3).

**FIGURE 2:**
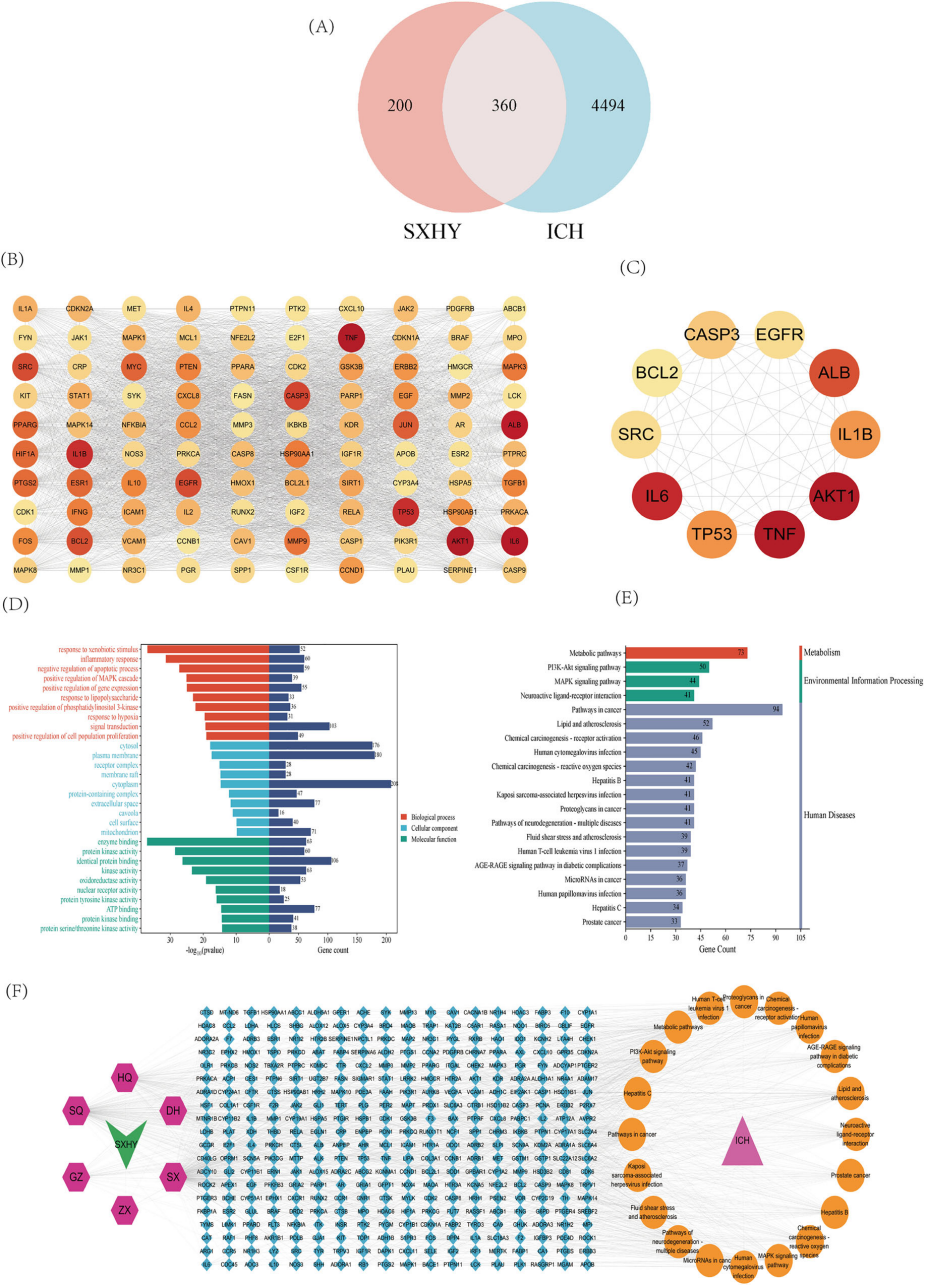
Network pharmacology results. (A) Venn diagram of overlapping targets between SXHY and ICH. (B) Topological analysis of PPI network nodes. (C) Core hub genes interaction network for SXHY in ICH treatment. (D) GO enrichment analysis for SXHY against ICH. (E) KEGG pathway enrichment of SXHY targets. (F) Drug–target–signaling pathway–disease network.

**TABLE 2:** Binding energies of key targets and active ingredient docking.

Molecule name	Docking score (kcal/mol)	Protein
3‐Methylcyclopentadecanone	−7.1	TNF‐α
Stigmasterol	−9.125	TNF‐α
Isoflavanone	−7.181	TNF‐α
Sitosterol	−9.07	TNF‐α
3‐Methylcyclopentadecanone	−7.214	IL‐1β
Stigmasterol	−8.964	IL‐1β
Isoflavanone	−7.978	IL‐1β
Sitosterol	−8.045	IL‐1β
3‐Methylcyclopentadecanone	−6.415	IL‐6
Stigmasterol	−8.57	IL‐6
Isoflavanone	−7.805	IL‐6
Sitosterol	−8.238	IL‐6

**FIGURE 3:**
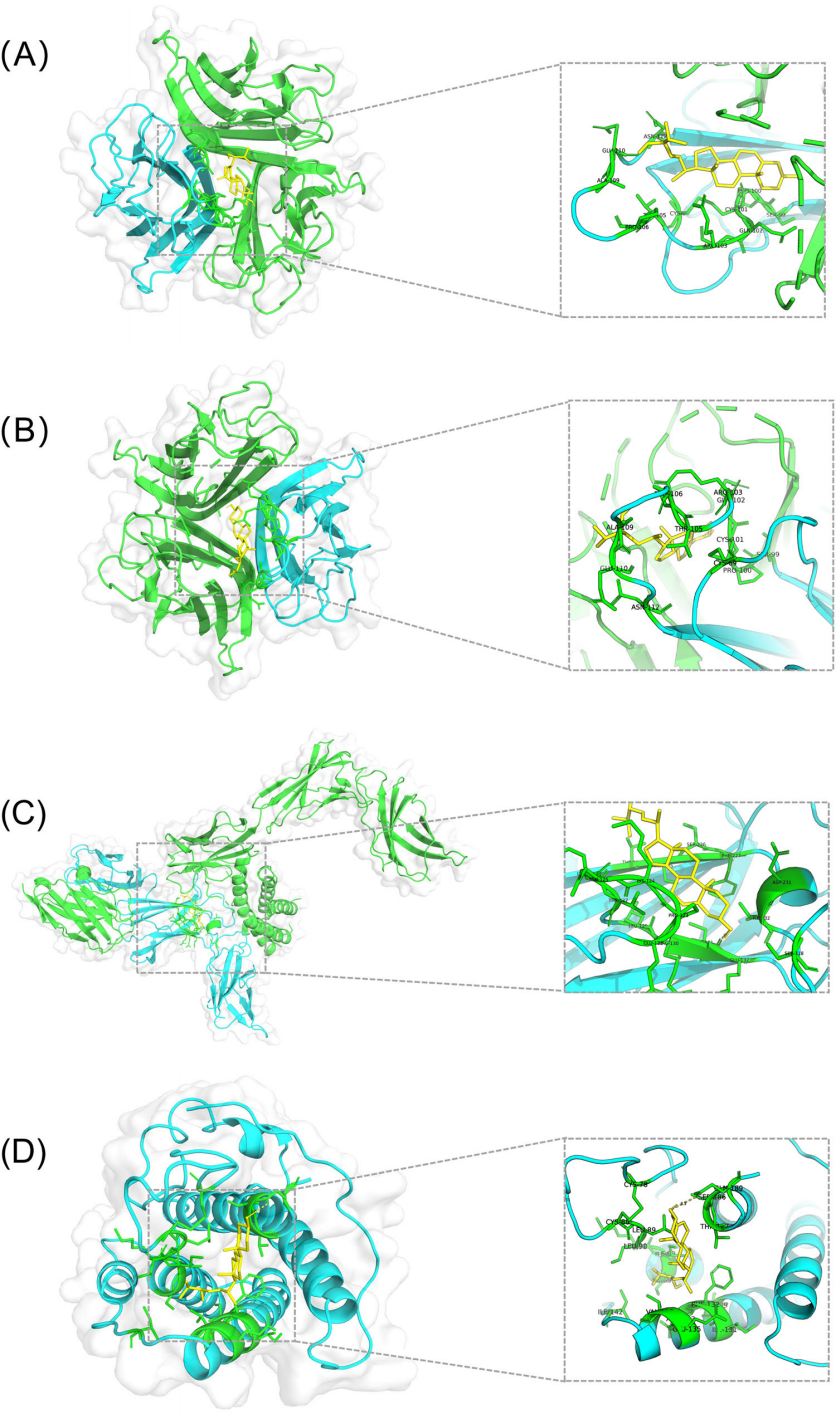
3D docking conformations of key SXHY components with core target proteins. (A) Stigmasterol with TNF‐α; (B) sitosterol with TNF‐α; (C) stigmasterol with IL‐1β; (D) stigmasterol with IL‐6.

### 3.2 Acute Toxicity Evaluation of SXHY

The acute toxicity assessment of SXHY revealed a favorable safety profile: (1) Survival: No mortality was observed during the 14‐day observation period, indicating the median lethal dose (LD^50^) was undeterminable; (2) Body weight: No statistically significant differences in body weight were detected between the SXHY and control groups (*p* > 0.05; Figure 4); (3) Organ indices: Heart, liver, spleen, lung, and kidney coefficients showed no significant abnormalities compared to the control group (*p* > 0.05; Figure 4); (4) Macroscopic examination: No pathological alterations (e.g., hemorrhage, necrosis) were found in major organs (heart, liver, spleen, lung, and kidney). Collectively, these results demonstrate that the MTD of SXHY exceeds 244.8 g crude drug/kg, suggesting a wide therapeutic window for clinical application.

**FIGURE 4:**
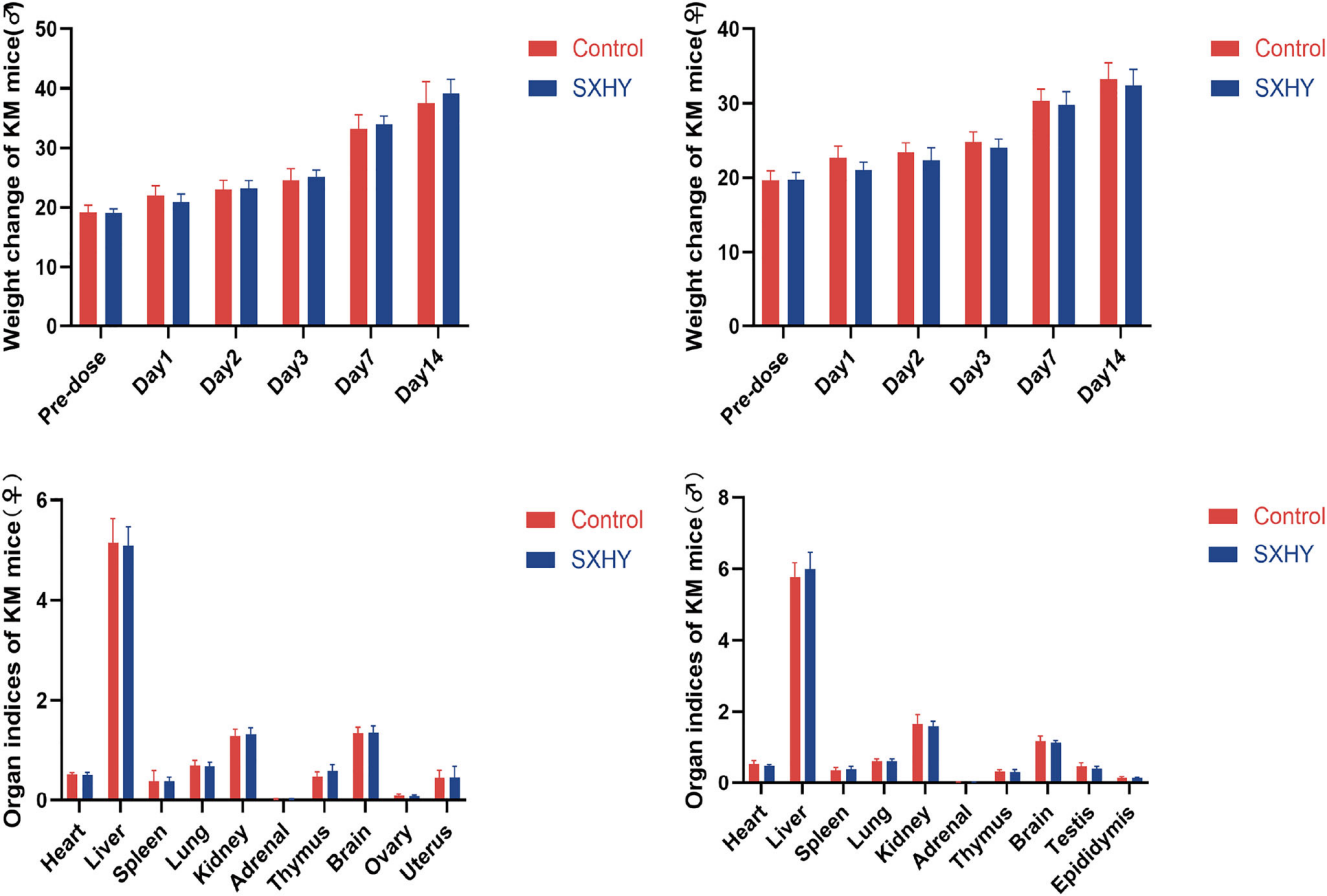
Safety trial of SXHY. (A) Male body weight. (B) Female body weight. (C) Female organ indices. (D) Male organ indices. Data are presented as mean ± SD (*n* = 10). No significant differences between SXHY and control groups (*p* > 0.05 by two‐way ANOVA).

### 3.3 Therapeutic Effects of SXHY on Neurological Recovery and Cerebral Protection in ICH Rats

Neurological function assessment showed no statistically significant between‐group differences in scores at 3 days post‐ICH (all *p* > 0.05). Within‐group comparisons from day 2 to day 3 showed significant score improvements in all groups: low‐dose (2.45 g crude drug/kg; 0.9 ± 0.6 to 0.1 ± 0.3, *p* < 0.01; 88.9% of animals improved), high‐dose (9.80 g crude drug/kg; 0.6 ± 0.5 to 0.1 ± 0.3, *p* < 0.01; 83.3%), medium‐dose (4.90 g crude drug/kg; 0.7 ± 0.5 to 0.2 ± 0.4, *p* < 0.01; 71.4%), model (1.0 ± 0.7 to 0.3 ± 0.5, *p* < 0.05; 70.0%), and simvastatin (0.8 ± 0.6 to 0.3 ± 0.5, *p* < 0.05; 62.5%).

For brain water content, the model group had a baseline value of 80.5 ± 1.0%. Compared to this, all SXHY dose groups and the simvastatin group exhibited statistically significant reductions: low‐dose (78.5 ± 1.8%, *p* < 0.05), medium‐dose (78.5 ± 1.2%, *p* < 0.05), high‐dose (78.0 ± 0.7%, *p* < 0.01), and simvastatin (78.1 ± 1.3%, *p* < 0.05).

Histopathological analysis using HE staining revealed that only the SXHY high‐dose group significantly decreased hematoma area to 0.35 ± 0.50 mm^2^ (vs. model group: 1.02 ± 0.93 mm^2^, ↓65.7%, *p* < 0.05), accompanied by marked improvements in perilesional inflammatory cell infiltration and edema (Figures 5 and 6). In contrast, the simvastatin group (0.43 ± 0.53 mm^2^) and the SXHY medium‐dose (0.57 ± 0.41 mm^2^) and low‐dose (0.80 ± 1.07 mm^2^) groups showed no statistically significant differences in hematoma area compared to the model group (*p* > 0.05).

**FIGURE 5:**
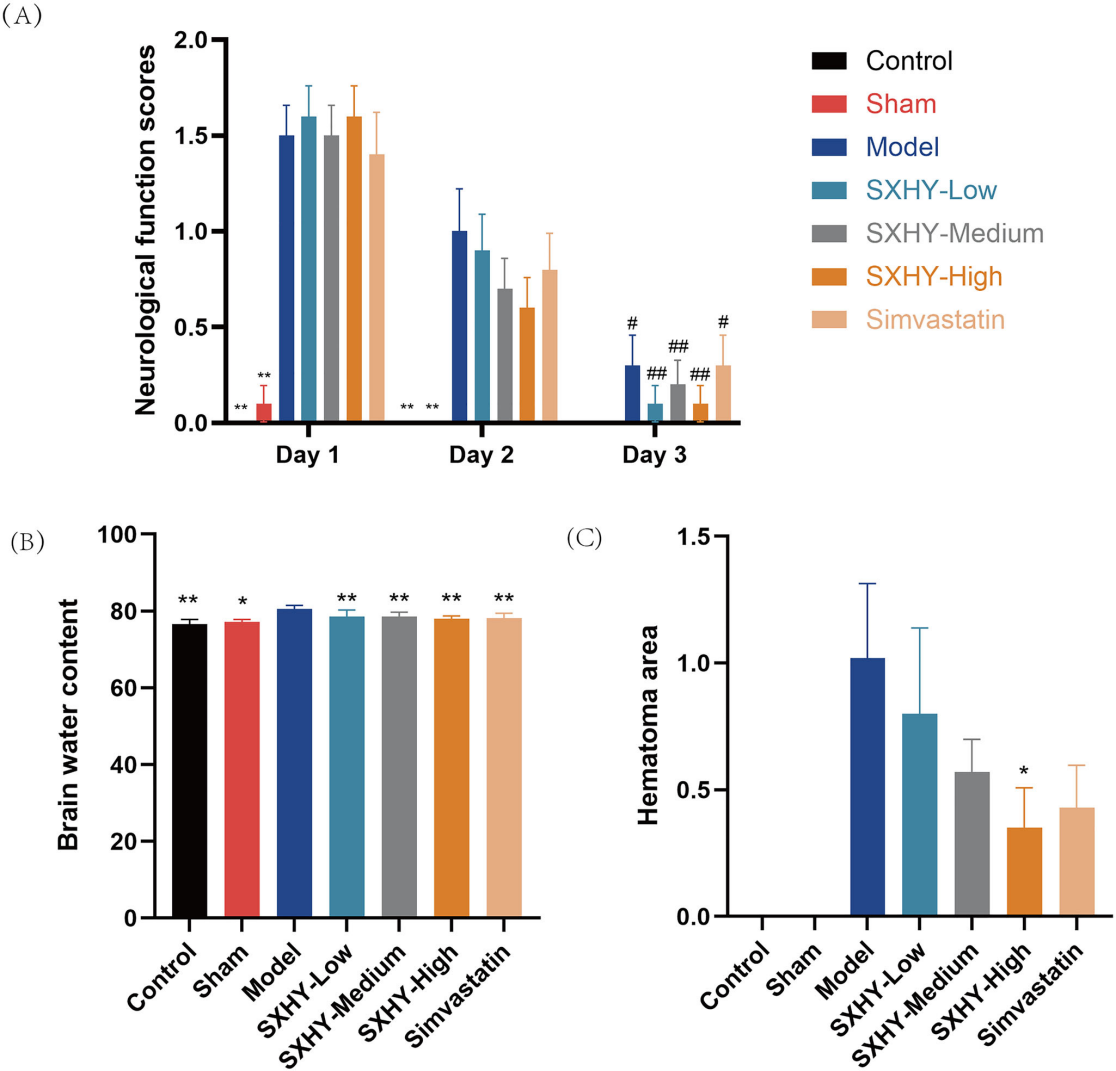
The effectiveness results of SXHY in treating ICH. (A)Effects of SXHY on neurological function scores in ICH rats. Data are presented as mean ± SD (*n* = 10). Intergroup comparisons (same time point): *p < 0.05, **p < 0.01 vs. the model group (one‐way ANOVA with Tukey's test). Intragroup comparisons (Day 3 vs. Day 2): ^#^ *p* < 0.05, ^##^ *p* < 0.01 (paired *t*‐test). Improvement magnitudes are calculated based on the following formula: [(D2 score – D3 score)/D2 score] × 100%. (B) Effects of SXHY on brain water content in ICH model rats. Data are presented as mean ± SD (*n* = 6). *p < 0.05, **p < 0.01 vs. model group (one‐way ANOVA with Tukey's test). (C) Effects of SXHY on hematoma area in ICH model rats. Data are presented as mean ± SD (*n* = 10). *p <0.05 vs. model group (one‐way ANOVA with Tukey's test). Hematoma area measured at day 7 post‐ICH induction. The reduction range = [(average value of the model group—average value of the treatment group)/average value of the model group] × 100%.

**FIGURE 6:**
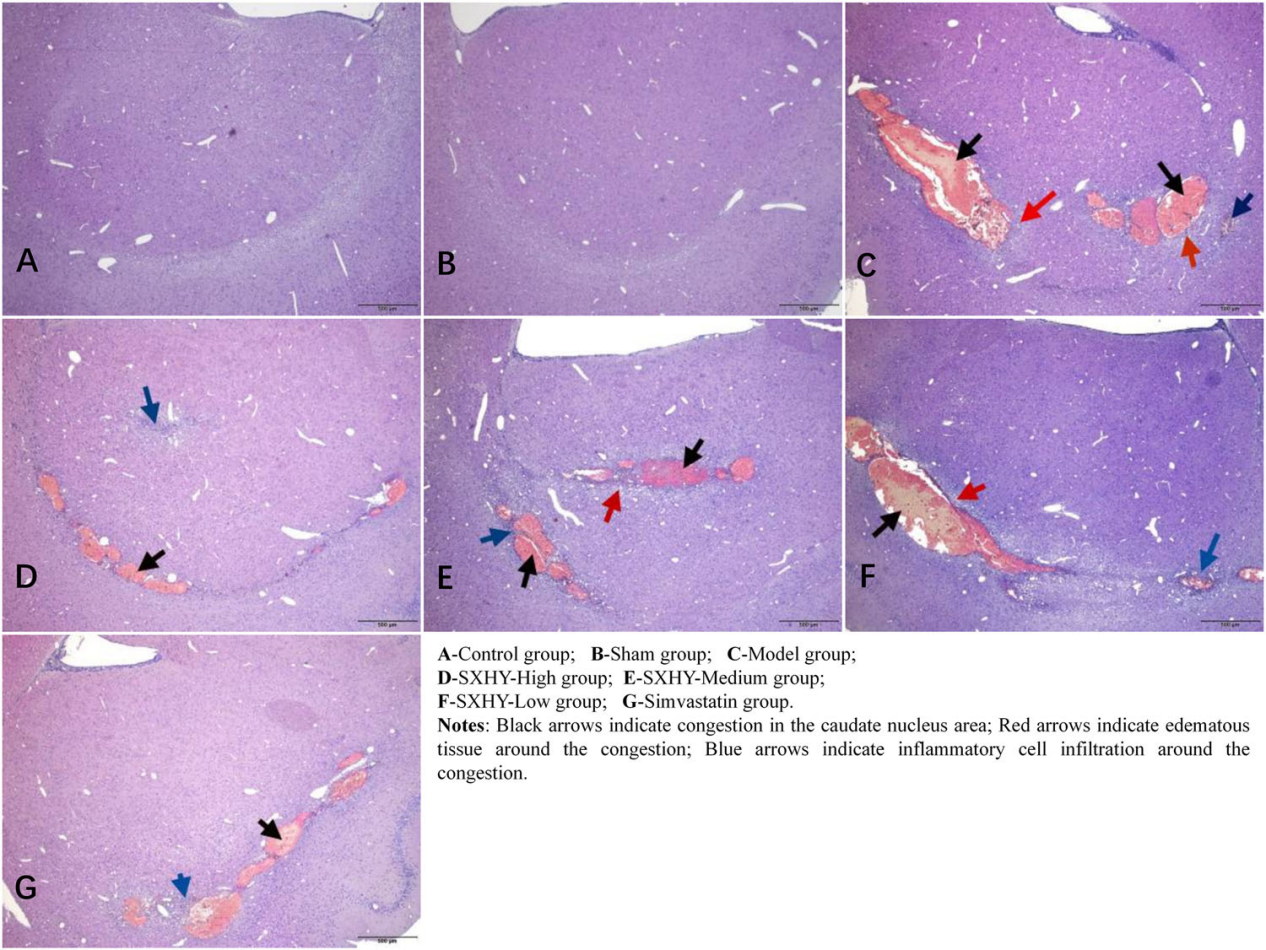
HE staining results of brain tissues in each group (40×).

Collectively, these findings demonstrate that SXHY improves neurological function score in ICH rats in a dose‐dependent manner, accelerates neurological recovery, and effectively reduces brain edema and tissue damage.

### 3.4 SXHY Modulates Oxidative Stress Markers in Brain Tissue Following ICH

Quantitative analysis demonstrated that SXHY significantly modulated oxidative stress parameters in the brain tissue of ICH rats, with differential effects on key antioxidant enzymes. SOD—a critical marker of antioxidant defense—exhibited significantly elevated activity in both the low‐dose (2.45 g crude drug/kg, 58.8 ± 8.7 U/mg protein) and high‐dose (9.80 g crude drug/kg, 68.7 ± 11.3 U/mg protein) SXHY groups compared to the model group (37.3 ± 14.1 U/mg protein), corresponding to 57.6% and 84.2% increases, respectively (both p < 0.01), while the medium‐dose group (4.90 g crude drug/kg, 60.7 ± 24.1 U/mg protein) showed a non‐statistically significant increasing trend. Regarding MDA—a biomarker of lipid peroxidation—all SXHY dose groups showed a consistent reduction in MDA levels compared to the model group (5.77 ± 0.69 nmol/mg protein), with the low‐dose group exhibiting an MDA level of 5.15 ± 0.52 nmol/mg protein; however, these reductions did not reach statistical significance (p > 0.05). Similarly, CAT activity exhibited modest increases in all SXHY groups (0.48–0.51 U/mg protein) compared to the model group (0.42 ± 0.10 U/mg protein), but no significant differences were observed (p > 0.05; Figure 7). These results indicate that SXHY enhances antioxidant defense in ICH rats primarily through significant upregulation of SOD activity, while the trends of SXHY in reducing MDA levels and increasing CAT activity warrant further validation using larger sample sizes or optimized dosage regimens.

**FIGURE 7:**
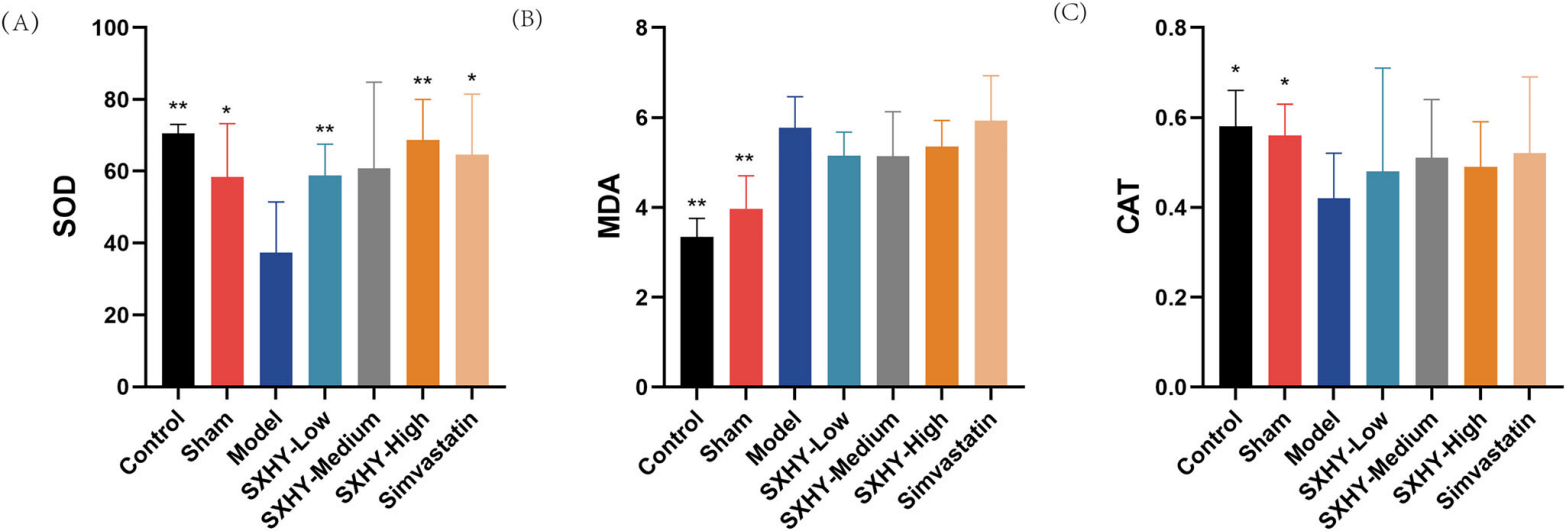
Effects of SXHY on oxidative stress markers in brain tissue of ICH model rats. (A) SOD. (B) MDA. (C) CAT. Data are presented as mean ± SD (*n* = 6). *p <0.05, **p <0.01 vs. model group (one‐way ANOVA with Tukey's post hoc test). SOD: superoxide dismutase; MDA: malondialdehyde; CAT: catalase.

### 3.5 SXHY Dose‐Dependently Modulates Inflammatory Cytokine Expression in LPS‐Stimulated BV‐2 Cells

Compared with the control group (1 ± 0), the model group (LPS 2 µg/mL) exhibited markedly elevated mRNA levels of the proinflammatory cytokines TNF‐α (9.03 ± 0.15), IL‐1β (6.62 ± 0.24), and IL‐6 (5.6 ± 0.52) (all ^###^ *p* < 0.001), demonstrating successful induction of an inflammatory response in BV‐2 cells. However, the mRNA level of the anti‐inflammatory cytokine IL‐10 (0.45 ± 0.45) did not show a significant change compared to the control group (*p* > 0.05).

Compared with the model group, SXHY exerted a dose‐dependent anti‐inflammatory effect: the SXHY low‐dose group (25 µg/mL) showed no significant alterations in TNF‐α (8.81 ± 0.14), IL‐1β (6.45 ± 0.06), IL‐6 (5.52 ± 0.54), or IL‐10 (1.26 ± 0.27) mRNA levels (all *p* > 0.05). In contrast, the SXHY medium‐dose group (50 µg/mL) significantly reduced TNF‐α (8.36 ± 0.37, *p < 0.05) and IL‐1β (5.4 ± 0.29, ***p < 0.001) while increasing IL‐10 (1.88 ± 0.38, **p < 0.01) mRNA levels, with no significant effect on IL‐6 (4.78 ± 0.2, *p* > 0.05). The SXHY high‐dose group (100 µg/mL) demonstrated the most potent regulatory effect on the inflammatory response, with significant decreases in TNF‐α (7.21 ± 0.31, ***p < 0.001), IL‐1β (4.23 ± 0.33, ***p < 0.001), and IL‐6 (3.07 ± 1.11, **p < 0.01) mRNA levels, alongside a marked increase in IL‐10 (2.21 ± 0.37, ***p < 0.001) mRNA.

Additionally, compared with the model group, the simvastatin group (10 µg/mL), a positive control, significantly inhibited TNF‐α (4.31 ± 0.1, ***p < 0.001) and IL‐1β (3.69 ± 0.21, ***p < 0.001) mRNA expression while increasing IL‐10 (1.79 ± 0.08, **p < 0.01) mRNA levels; however, it exerted no significant effect on IL‐6 (3.95 ± 0.88, *p* > 0.05). Compared with the simvastatin group, the SXHY high‐dose group exhibited a significantly higher TNF‐α mRNA level (7.21 ± 0.31, ^@@@^ *p* < 0.001) but no significant differences in IL‐1β (4.23 ± 0.33, *p* > 0.05), IL‐6 (3.07 ± 1.11, *p* > 0.05), or IL‐10 (2.21 ± 0.37, *p* > 0.05) mRNA levels (Figure 8).

**FIGURE 8:**
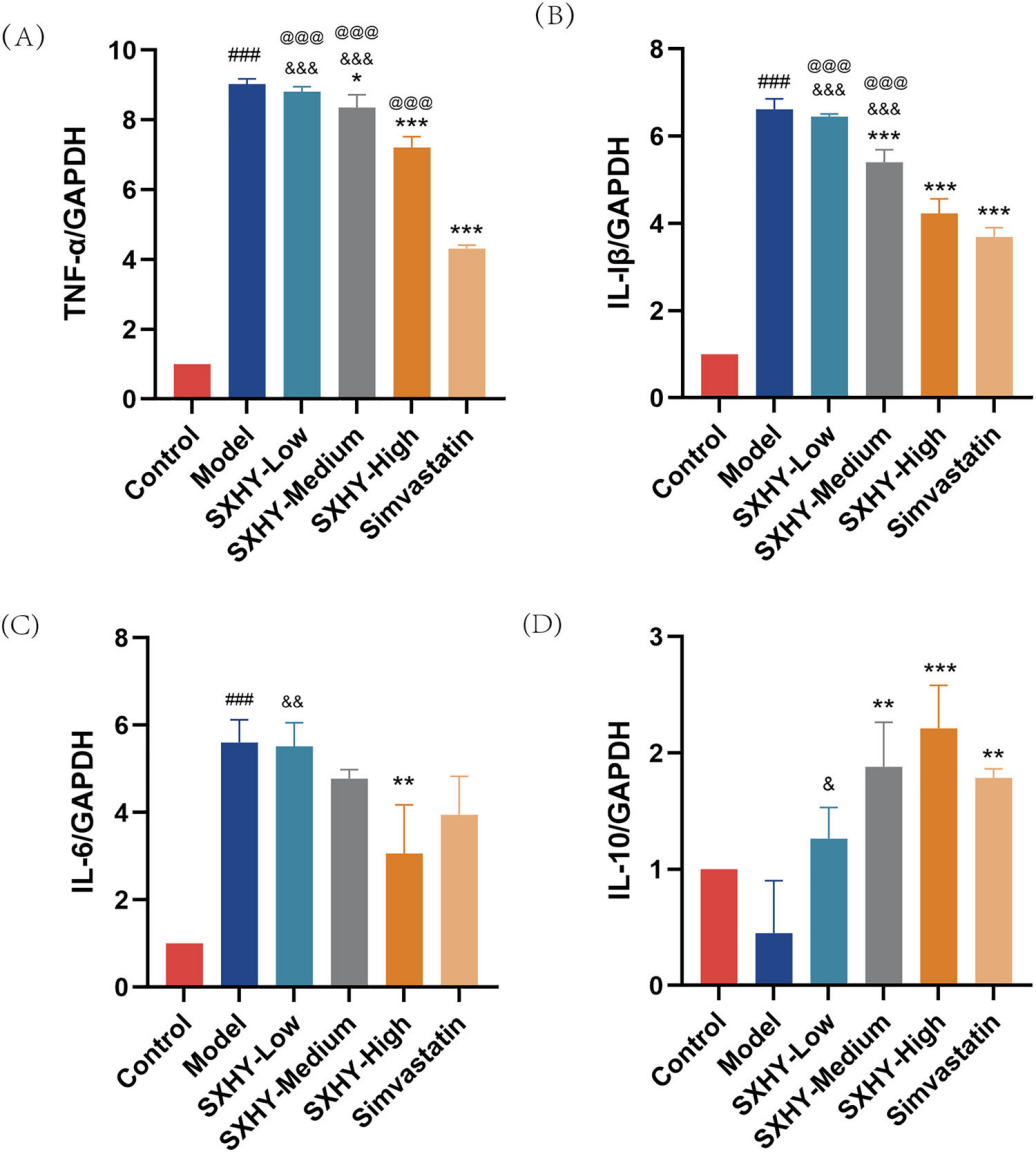
Changes in mRNA expression. (A) TNF‐α. (B) IL‐1β. (C) IL‐6. (D) IL‐10. Data are presented as mean ± SD (*n* = 3). Comparison between model group and control group: ^##^ *p* < 0.01, ^###^ *p* < 0.001; Comparison between drug groups and model group: *p < 0.05, **p < 0.01, ***p < 0.001. Comparison between drug group and simvastatin group: ^@@@^ *p* < 0.001.

### 3.6 SXHY Modulates PI3K/AKT Signaling in LPS‐Stimulated BV‐2 Cells

To investigate whether SXHY regulates the PI3K/AKT pathway, we quantified total and phosphorylated PI3K and AKT by Western blotting (Figure 9). LPS stimulation (2 µg/mL) significantly increased total PI3K/GAPDH (1.87 ± 0.11 vs. 1.00 ± 0, ^###^ *p* < 0.001) and total AKT/GAPDH (2.22 ± 0.13 vs. 1.00 ± 0, ^###^ *p* < 0.001) compared with the control group. Phosphorylated PI3K and AKT were also elevated, but to a lesser extent (*p*‐PI3K/GAPDH: 1.13 ± 0.06 vs. 1.00 ± 0, ^##^ *p* < 0.01; *p*‐AKT/GAPDH: 1.29 ± 0.07 vs. 1.00 ± 0, ^###^ *p* < 0.001). Consequently, the phosphorylation‐to‐total ratios in the model group were lower than the normalized control value of 1.00 (*p*‐PI3K/PI3K: 1.13/1.87 = 0.60; *p*‐AKT/AKT: 1.29/2.22 = 0.58), indicating that LPS did not increase the proportion of phosphorylated PI3K or AKT under these conditions.

**FIGURE 9:**
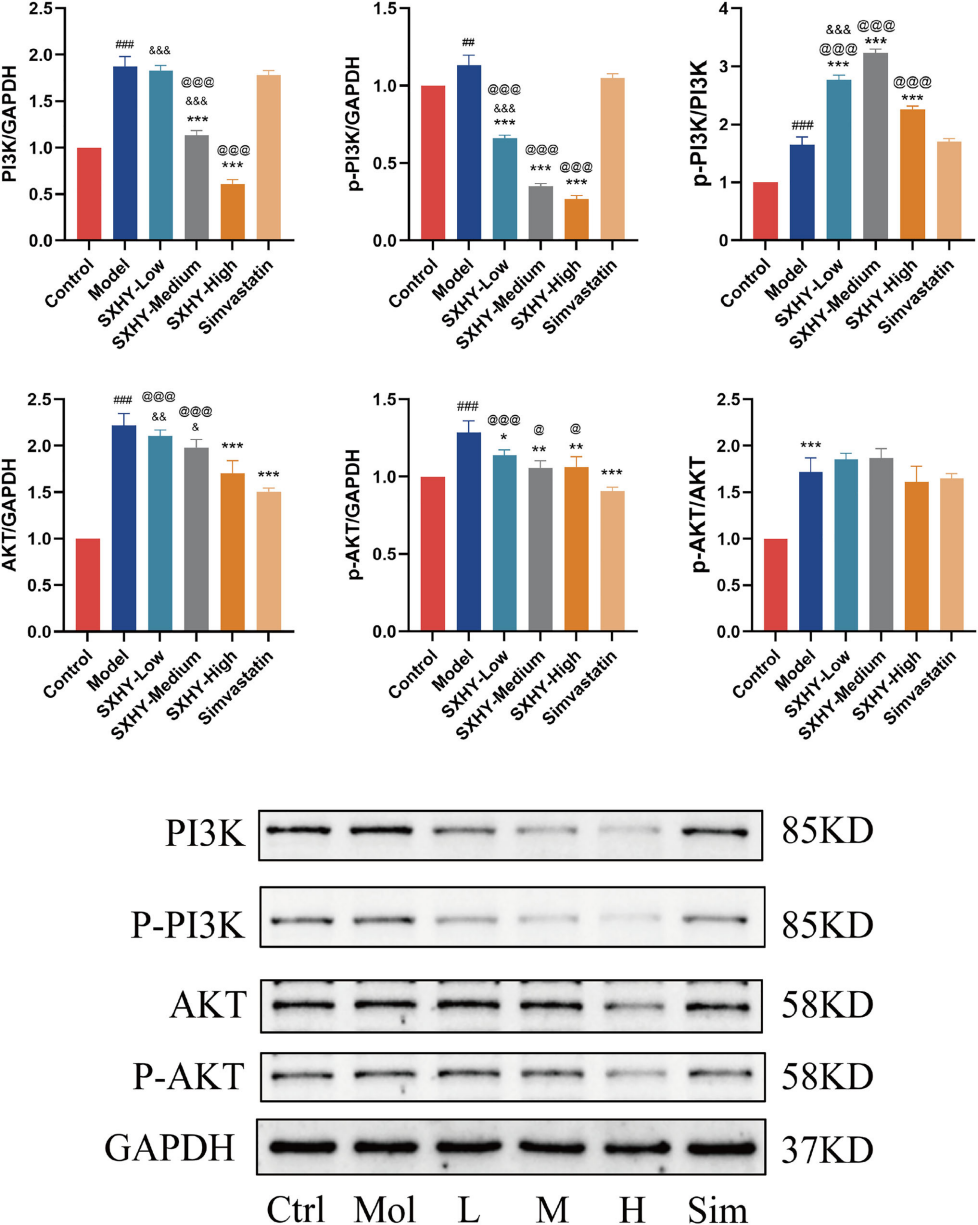
WB experimental results. Data are presented as mean ± SD (*n* = 3 independent experiments). The phosphorylation‐to‐total ratios were calculated as phosphorylated protein divided by total protein. Between‐group comparisons were performed by one‐way ANOVA with Tukey's post hoc test. ^#^ *p* < 0.05, ^##^ *p* < 0.01, ^###^ *p* < 0.001 vs. Control; * *p* < 0.05, **p < 0.01, ***p < 0.001 vs. Model; ^@^ *p* < 0.05, ^@@^ *p* < 0.01, ^@@@^ *p* < 0.001 vs. Simvastatin.

SXHY dose‐dependently decreased *p*‐PI3K/GAPDH (low dose: 0.66 ± 0.02; medium dose: 0.35 ± 0.02; high dose: 0.27 ± 0.02; all ***p < 0.001 vs. model). Total PI3K/GAPDH was significantly reduced at the medium (1.13 ± 0.05, ***p < 0.001 vs. model) and high (0.61 ± 0.05, ***p < 0.001 vs. model) doses, whereas the low dose (1.83 ± 0.05) showed no significant change relative to the model group. The recalculated *p*‐PI3K/PI3K ratios were 0.36, 0.31, and 0.44 for the low, medium, and high doses, respectively, compared with 0.60 in the model group. For AKT, high‐dose SXHY reduced total AKT/GAPDH (1.71 ± 0.14, ***p < 0.001 vs. model) and *p*‐AKT/GAPDH (1.06 ± 0.07, **p < 0.01 vs. model), with a *p*‐AKT/AKT ratio of 0.62 vs. 0.58 in the model group. The low and medium doses also decreased *p*‐AKT/GAPDH (1.14 ± 0.03 and 1.06 ± 0.05, respectively; *p < 0.05 and **p < 0.01 vs. model) but had limited effects on total AKT/GAPDH. Simvastatin (20 µg/mL) reduced total AKT/GAPDH (1.50 ± 0.04) and *p*‐AKT/GAPDH (0.91 ± 0.03) but did not significantly alter total PI3K/GAPDH or p‐PI3K/GAPDH. The p‐PI3K/PI3K ratio for simvastatin was 0.59, comparable to the model group.

Collectively, these data indicate that SXHY primarily suppresses PI3K phosphorylation rather than activating PI3K/AKT signaling in LPS‐stimulated BV‐2 cells. The reduction in p‐PI3K/GAPDH occurred in a dose‐dependent manner, while changes in total PI3K and AKT protein levels were dose‐ and protein‐specific. These findings suggest that modulation of PI3K/AKT signaling by SXHY may contribute to its anti‐inflammatory effects, although the downstream consequences require further investigation.

### 3.7 Regulation of Inflammatory Cytokine mRNA Expression by SXHY: Dependence on the PI3K/AKT Pathway

To verify whether SXHY's regulation of inflammatory cytokines depends on the PI3K/AKT pathway, we established three treatment groups: normal (no inhibitors), PI‐103 (a PI3K inhibitor), and YS‐49 (an AKT inhibitor). We quantified mRNA expression of TNF‐α, IL‐1β, IL‐6, and IL‐10 (Figure 10). The normal group's model subgroup (treated with LPS at 2 µg/mL) exhibited TNF‐α, IL‐1β, and IL‐6 mRNA levels of 9.03 ± 0.15, 6.62 ± 0.24, and 5.62 ± 0.52, respectively. Its SXHY‐High subgroup (100 µg/mL) showed corresponding levels of 7.21±0.31, 4.23 ± 0.33, and 3.07 ± 1.11 for these cytokines. Compared to the normal group model subgroup, the PI‐103 group model subgroup exhibited significantly lower TNF‐α (3.67 ± 0.38, ^##^ *p <*0.01), IL‐1β (3.53 ± 0.07, ^#^ *p <* 0.05), and IL‐6 (0.16 ± 0.18, ^###^ *p <* 0.001) mRNA levels. Similarly, the YS‐49 group model subgroup showed significantly reduced TNF‐α (4.58 ± 0.4, ^##^ *p <* 0.01), IL‐1β (3.84 ± 0.35, ^#^ *p <* 0.05), and IL‐6 (1.76 ± 0.19, ^###^ *p <* 0.001) mRNA levels relative to the normal group model subgroup. In the PI‐103 group, SXHY inhibited inflammatory cytokines in a dose‐dependent manner: compared to the corresponding normal group SXHY‐High subgroup, the PI‐103+SXHY‐High subgroup exhibited significantly lower TNF‐α (1.1 ± 0.24, ***p < 0.001), IL‐1β (1.17 ± 0.15, ***p < 0.001), and IL‐6 (0.05 ± 0.04, *p < 0.05) mRNA levels. In the YS‐49 group, compared to the corresponding normal group SXHY‐High subgroup, the YS‐49+SXHY‐High subgroup also showed significantly reduced TNF‐α (2.45 ± 0.3, ***p < 0.001), IL‐1β (2.31 ± 0.11, ***p < 0.001), and IL‐6 (0.86 ±0.09, *p < 0.05) mRNA levels. Notably, the PI‐103 group SXHY‐High subgroup exhibited consistently lower inflammatory cytokine mRNA levels than the corresponding YS‐49 group subgroup. IL‐10 mRNA expression showed no consistent trend across groups and was not significantly modulated by SXHY or the inhibitors. These results suggest that the regulation of inflammatory cytokines—including TNF‐α, IL‐1β, and IL‐6—by SXHY depends on the PI3K/AKT pathway. Figure 11 is a potential mechanism diagram for this article.

**FIGURE 10:**
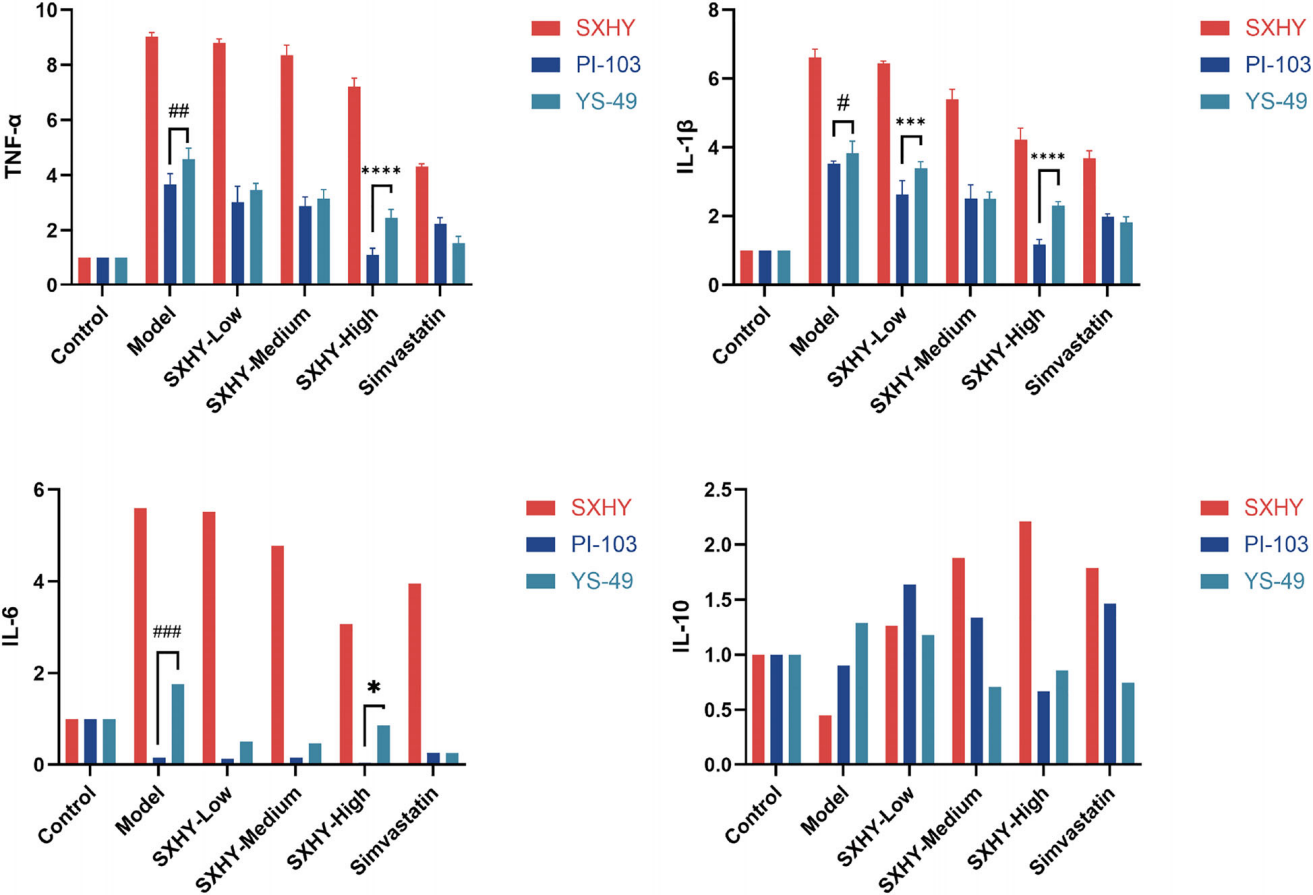
Effects of normal, PI‐103, and YS‐49 on IL‐1β, TNF‐α, IL‐6, and IL‐10mRNA expression. Data are presented as mean ± standard deviation (SD) (*n* = 3). ^#^ *p* <0.05, ^##^ *p* < 0.01, ^###^ *p* < 0.001: Significant differences between the model subgroup (LPS 2 µg/mL) of PI‐103/YS‐49 groups and the model subgroup of the normal group (baseline LPS‐induced inflammation). *p < 0.05, **p < 0.01, ***p < 0.001: Significant differences between the drug‐treated subgroups (SXHY‐low/medium/high, simvastatin) of PI‐103/YS‐49 groups and the corresponding drug‐treated subgroups of the normal group (e.g., PI‐103+SXHY‐high vs. normal + SXHY‐high).

**FIGURE 11:**
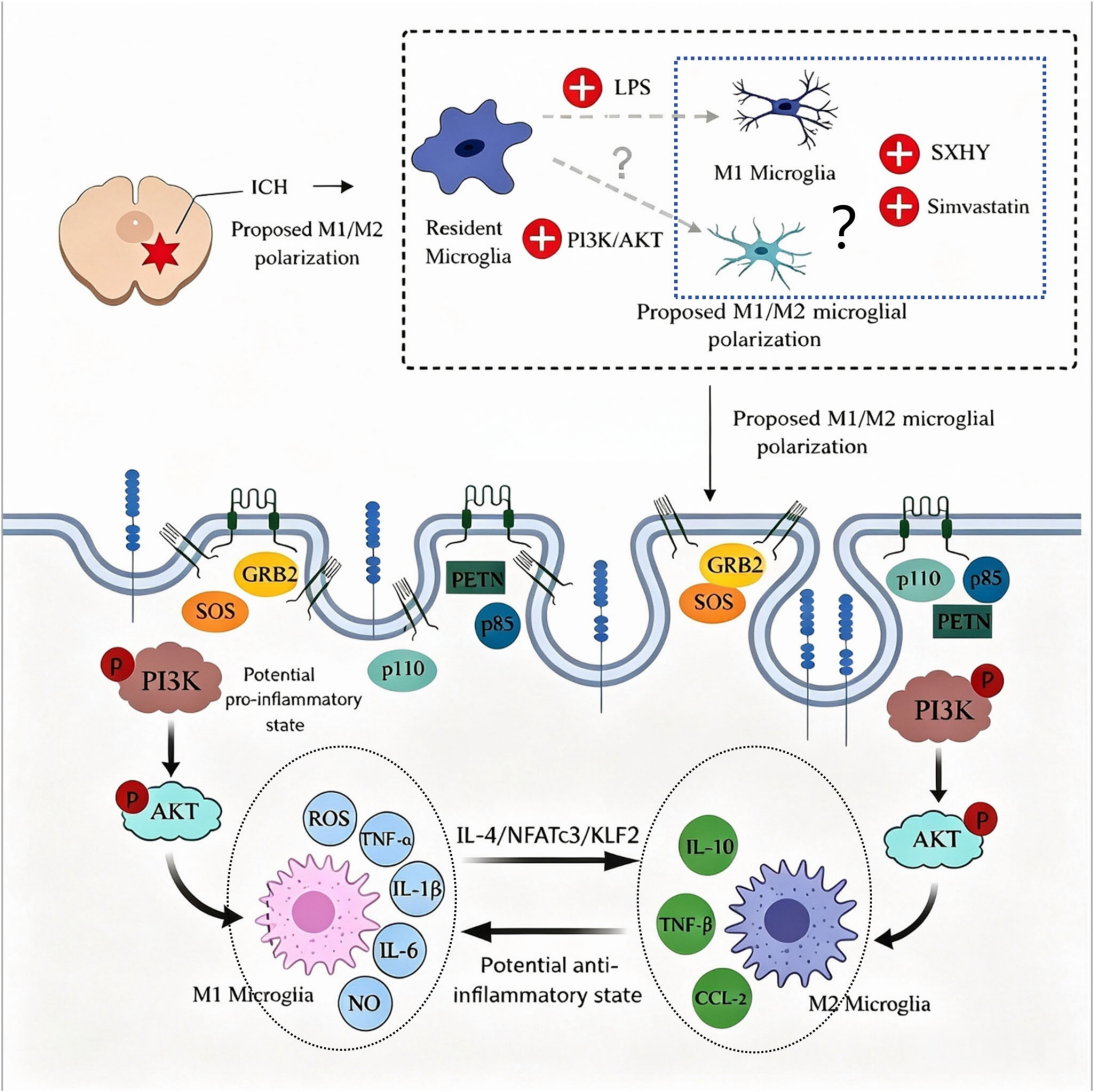
Proposed mechanism by which SXHY alleviates ICH‐induced neuroinflammation via modulation of PI3K/AKT signaling in microglia.

## 4 Discussion

This research represents the inaugural elucidation of the molecular mechanism through which SXHY—a TCM formulation—ameliorates the pathological progression of ICH. Specifically, SXHY suppresses neuroinflammation by regulating the PI3K/AKT pathway, thereby alleviating post‐ICH cerebral edema and promoting hematoma absorption. These findings thus underscore the convergent evidence derived from network pharmacology prediction coupled with rigorous experimental validation.

The central finding of this study is that SXHY markedly alleviates post‐ICH cerebral edema and promotes hematoma absorption by suppressing the PI3K phosphorylation‐mediated inflammatory cascade. Animal experiments showed that high‐dose SXHY reduced brain water content from 80.5% (model group) to 78.0% (*p <* 0.01) and decreased hematoma area by 65.7% (*p <* 0.05). These effects were attributed to modulation of PI3K/AKT signaling, particularly reduced PI3K phosphorylation, thereby attenuating downstream inflammatory cascades (e.g., proinflammatory cytokine release). Furthermore, although no statistically significant between‐group difference was observed at postoperative day 3, the observed proportions of animals with improved neurological scores were 88.9% (low‐dose SXHY), 83.3% (high‐dose SXHY), 70.0% (model), and 62.5% (simvastatin). This suggests that SXHY may accelerate the process of neurological function recovery, a trend that aligns with previous studies (Song et al. 2024; Guo et al. 2024) reporting that TCM formulations containing *Moschus* (musk) and *Panax Notoginseng* (sanqi) enhance neurological function recovery post‐ICH. Notably, the high‐dose SXHY group exhibited a greater improvement in hematoma area (65.7%) compared to the simvastatin group (57.8%). This may be directly attributed to the role of *Notoginseng* saponins (a key component of SXHY) in enhancing the clearance of inflammatory mediators via the meningeal lymphatic system. As a classic lipid‐lowering agent, simvastatin primarily exerts its effects by modulating lipid metabolism, with limited direct impact on hematoma absorption—likely explaining the lack of statistically significant improvement in hematoma area in the simvastatin group.

Network pharmacology findings were consistent with experimental validation. Analysis revealed that 98 bioactive components of SXHY target 360 ICH‐related proteins, with the PI3K/AKT pathway identified as the core signaling axis regulating ICH pathophysiology. This prediction was robustly validated in cellular experiments using an LPS‐induced BV‐2 microglial cell model. The high‐dose SXHY group (100 µg/mL) significantly reduced p‐AKT/GAPDH (1.06 ± 0.07 vs. 1.29 ± 0.07 in the model group, **p < 0.01) and total AKT/GAPDH (1.71 ± 0.14 vs. 2.22 ± 0.13 in the model group, ***p < 0.001). The p‐AKT/AKT ratio was 0.62 in the high‐dose group vs. 0.58 in the model group and did not indicate reduced AKT phosphorylation relative to total AKT. High‐dose SXHY also markedly suppressed the mRNA expression of proinflammatory cytokines TNF‐α (7.21 ± 0.31 vs. 9.03 in the model group, ***p < 0.001; 20.15% decrease) and IL‐6 (3.07 ± 1.11 vs. 5.6 ± 0.52 in the model group, **p < 0.01; 45.18% decrease). Furthermore, molecular docking results uncovered a direct targeting mechanism of SXHY: stigmasterol (a key bioactive component) exhibited a strong binding affinity with TNF‐α, with a binding energy of −9.125 kcal/mol. However, in cellular experiments, TNF‐α mRNA expression in the high‐dose SXHY group remained significantly higher than in the simvastatin group. This suggests that the direct targeting effect of stigmasterol may be attenuated by the antagonistic actions of other components, or that the “multi‐component, multi‐target” synergistic effect of SXHY was not fully exploited (e.g., no significant effects at low doses). As a key regulator of multiple pathological processes in ischemic stroke, the PI3K/AKT pathway exhibits intimate mechanistic connections with disease progression (Liu et al. 2023; Liu et al. 2025; Lu et al. 2022). However, research on modulating this pathway for ICH treatment remains limited (Zhu et al. 2022; Crilly et al. 2021). SXHY exerts a dual mechanism: it modulates PI3K/AKT signaling, primarily by reducing PI3K phosphorylation, and enhances anti‐inflammatory effects by directly binding to inflammatory mediators (e.g., TNF‐α), consistent with TCM's “multi‐component, multi‐target” synergy. This “pathway modulation + direct targeting” dual mode of action provides novel evidence for the involvement of the PI3K/AKT pathway in TCM‐based ICH therapy and further exemplifies the “holistic regulation and synergistic enhancement” advantages of TCM formulations.

The efficacy and safety profile of SXHY underscores its potential for clinical translation. High‐dose SXHY showed greater neurological function score improvement (83.3% vs. 62.5% in the simvastatin group) and uniquely upregulated IL‐10 mRNA—unlike simvastatin, which only suppressed proinflammatory cytokines. This balanced immunomodulatory effect is likely attributable to the synergistic actions of the TCM components: *Moschus* (musk) suppresses TNF‐α/RIPK1 necroptosis (downregulating AQP4/HIF‐1α) to alleviate neuroinflammation and edema, *Panax Notoginseng* (sanqi) promotes hematoma absorption, and *Astragali* Radix (huangqi) enhances antioxidant capacity. Safety assessments revealed that the safety threshold of SXHY in mouse acute toxicity studies (244.8 g crude drug/kg)—converted to a rat equivalent dose of 105.5 g crude drug/kg via body surface area normalization (Reagan‐Shaw et al. 2008)—was 10.8‐fold higher than the maximum pharmacodynamic dose (9.80 g crude drug/kg), complying with OECD guidelines for preclinical safety margins (OECD 2008). Furthermore, the recommended clinical dose (0.816 g crude drug/kg) was well below the conventional drug safety limit (U.S. Food and Drug Administration 2005). These findings establish SXHY's therapeutic promise for ICH.

SXHY exhibited a hierarchical, upstream‐prioritized pattern in regulating oxidative stress in the brain tissues of ICH rats. The high‐dose SXHY group (9.80 g crude drug/kg) significantly elevated SOD activity (68.7 ± 11.3 vs. 37.3 ± 14.1 U/mg protein in the model group; *p <* 0.01). However, neither CAT activity nor MDA levels displayed statistically significant alterations. This suggests SXHY prioritizes upstream O_2_^−^ clearance (SOD‐mediated) during oxidative stress but does not concurrently regulate midstream H_2_O_2_ decomposition (CAT‐dependent) or terminal MDA formation. In the context of the classic oxidative stress metabolic pathway (O_2_^−^ → SOD → H_2_O_2_ → CAT → H_2_O + O_2_), the lack of a significant increase in CAT activity may result in H_2_O_2_ accumulation, which in turn impairs MDA reduction. This observation may be attributed to a small sample size or insufficient treatment duration (e.g., long‐term changes in CAT activity or MDA levels were not assessed) and warrants further investigation. Nevertheless, the significant elevation in SOD activity suggests that SXHY possesses potential for early antioxidant intervention, providing experimental support for its clinical investigation in post‐ICH oxidative stress.

We acknowledge several limitations of this study. First, interspecies disparities in ICH pathophysiology (e.g., hematoma evolution patterns, inflammatory cascades) and xenobiotic metabolism necessitate cautious extrapolation of our rat model‐derived findings to human clinical scenarios. Second, the BV‐2 single‐cell culture system cannot recapitulate neuron–microglia crosstalk in the ICH microenvironment, potentially limiting the comprehensiveness of our mechanistic findings. Third, the brain distribution of the 98 bioactive components in SXHY has not been quantified, obscuring the key pharmacodynamic material basis of its efficacy. Fourth, the downstream effectors of the PI3K/AKT pathway (e.g., mTOR, NF‐κB) have not been fully elucidated, limiting the depth of our mechanistic understanding. Finally, we acknowledge that MAPK and AGE‐RAGE signaling, though enriched in our network analysis, were not experimentally validated in the current study. However, this does not exclude their potential synergistic contributions to SXHY's therapeutic effects. Our decision to focus on PI3K/AKT as the principal axis was based on its network hub status and overarching regulation of multiple ICH‐related pathologies, leaving the exploration of other pathways as independent directions for subsequent research. In addition, future translational research necessitates clinical trials to corroborate the therapeutic potential of SXHY in human populations, employ neuron–microglia co‐culture models to mimic neuroinflammation, use UPLC‐Q‐TOF/MS to quantify the biodistribution of SXHY components, and apply conditional gene knockout models to dissect the downstream signaling of the PI3K/AKT pathway.

In conclusion, SXHY mitigates post‐ICH neuroinflammation and oxidative damage through multitarget modulation of the PI3K/AKT pathway. Its validated efficacy and wide safety margin (10.8‐fold) strongly support its clinical translation for ICH treatment. Future work can build upon these findings to optimize clinical dosing regimens and explore the potential for combination therapy with other anti‐ICH agents.

## Author Contributions

**Yuan Yuan** and **Chen Meijuan** conceived and designed the study. **Yuan Rukui**, **Wang Mingli**, and **Li Chun** performed the experiments and analyzed the data. Yuan Rukui wrote the manuscript. All authors reviewed and approved the final version.

## Funding

This study was supported by the Central Finance Traditional Chinese Medicine Inheritance and Development Subsidy Project (Subproject: Evidence‐Based Capacity Enhancement Project of Traditional Chinese Medicine, No. 2100601), the Science and Technology Project of the Sichuan Administration of Traditional Chinese Medicine (No. 2024MS210), and the Luzhou Science and Technology Plan Project (No. 2022SYF37).

## Conflicts of Interest

The authors declare no conflicts of interest.

## Data Availability

The data that support the findings of this study are available from the corresponding author upon reasonable request.
